# FAPI PET/CT in the Diagnosis of Abdominal and Pelvic Tumors

**DOI:** 10.3389/fonc.2021.797960

**Published:** 2022-01-04

**Authors:** Tianshuo Yang, Long Ma, Haodong Hou, Feng Gao, Weijing Tao

**Affiliations:** ^1^ Department of Nuclear Medicine, The Affiliated Huaian No.1 People’s Hospital of Nanjing Medical University, Huaian, China; ^2^ Department of Medical Imaging, Jinling Hospital, Medical School of Nanjing University, Nanjing, China; ^3^ Key Laboratory for Experimental Teratology of the Ministry of Education and Center for Experimental Nuclear Medicine, School of Basic Medical Sciences, Cheeloo College of Medicine, Shandong University, Jinan, China

**Keywords:** ^68^Ga-FAPI-04, ^18^F-FDG, positron emission tomography computed tomography (PET/CT), abdominal tumors, pelvic tumors, radiotherapy

## Abstract

Positron emission tomography/computed tomography (PET/CT) with ^18^F-fluorodeoxyglucose (^18^F-FDG) is currently a standard imaging examination used in clinical practice, and plays an essential role in preoperative systemic evaluation and tumor staging in patients with tumors. However, ^18^F-FDG PET/CT has certain limitations in imaging of some tumors, like gastric mucus adenocarcinoma, highly differentiated hepatocellular carcinoma, renal cell carcinoma, and peritoneal metastasis. Therefore, to search for new tumor diagnosis methods has always been an important topic in radiographic imaging research. Fibroblast activation protein (FAP) is highly expressed in many epithelial carcinomas, and various isotope-labelled fibroblast activation protein inhibitors (FAPI) show lower uptake in the brain and abdominal tissues than in tumor, thus achieving high image contrast and good tumor delineation. In addition to primary tumors, FAPI PET/CT is better than FDG PET/CT for detecting lymph nodes and metastases. Additionally, the highly selective tumor uptake of FAPI may open up new application areas for the non-invasive characterization, staging of tumors, as well as monitoring tumor treatment efficacy. This review focuses on the recent research progress of FAPI PET/CT in the application to abdominal and pelvic tumors, with the aim of providing new insights for diagnostic strategies for tumor patients, especially those with metastases.

## 1 Introduction

In recent years, tumor morbidity and mortality have increased rapidly. According to the statistics, 19.3 million new cancer cases and nearly 10 million cancer deaths occurred in 2020 (1). In the abdominal and pelvic cavity, colorectal cancer, liver cancer, gastric cancer, and prostate cancer all have high morbidity and mortality (2–5). Therefore, a large amount of clinical and basic research is needed to improve the detection and treatment of tumors (6). Early diagnosis and accurate assessment of tumors have important implications for treatment decisions and prognosis (7).

“Tumors” comprise cancer cells and stromal cells: cancer cells are undifferentiated, while stromal cells consist of fibroblasts, vascular endothelial cells, and immune cells (8). At present, PET/CT is a commonly used imaging method for the preoperative systemic evaluation and staging of malignant tumors and the glucose analog, fluorodeoxyglucose (FDG) is the most commonly used radioactive tracer for detecting malignant tumors on PET/CT (9–14). However, FDG has weak efficacy for tumor delineation and the identification of metastatic lesions (15, 16, 17), making it difficult to distinguish between inflammation and malignancy on FDG PET/CT (7, 18–20).

Recently, the tumor microenvironment (TME) has attracted increasing attention as a target for tumor therapy. The TME occupies a significant fraction of the tumor volume. For example, in pancreatic cancer, TME can form up to 80% of tumor blocks (21). Cancer-associated fibroblasts (CAFs), existing in most of the stroma of solid tumors, are essential components of the TME (22, 23). CAFs are thought to originate from various cells, including fibroblasts, adipocytes, and epithelial cells (24, 25). Fibroblast activation protein (FAP) was initially discovered in the malignant cells of many sarcomas by Rettig et al. in 1988 (26). FAP is a serine protease that is barely expressed in the matrix of healthy tissues (27, 28). However, in many epithelial cancers, particularly connective tissue, ovarian, pancreatic, and hepatocellular cancers, CAF is observed to show high expression (22, 29–32). CAFs differ from normal fibroblasts in that they show a relatively higher tumor-specific expression of FAP, which is expressed in over 90% of human epithelial carcinomas but is almost absent in normal adult tissues (33–35). Hypoxia is an influential factor in the induction of FAP expression in CAF (36). Currently, fibroblast activation protein inhibitors (FAPI) have been developed as anticancer agents (37), and most tumors show high uptake rates and image contrast on FAPI PET/CT (38–41), which is helpful for non-invasive qualitative analysis of tumor, staging examinations, and radioligand therapy (18, 19, 42–45). Therefore, in this review article, we focus on the recent progress of FAPI PET/CT for the examination of abdominal and pelvic tumors.

## 2 Organs of the Abdominal Cavity

### 2.1 Canal Organs

#### 2.1.1 Stomach

Globally, gastric cancer is one of the most common malignancies, with more than one million new cases and an estimated 769 000 deaths in 2020, and is in fifth place for global morbidity and fourth for mortality (1). The risk factors for gastric cancer include *Helicobacter pylori* infection, age, high salt intake, and low intake of fruit and vegetables (1, 46). Early detection and early prevention are essential measures to reduce mortality from gastric cancer, and are very important for its treatment and prognosis (4, 46, 47). ^18^F-FDG PET/CT has poor diagnostic ability for gastric signet-ring cell carcinoma, gastric mucus adenocarcinoma, and non-interstitial diffuse gastric cancer (12), which is because these histological types of gastric cancer can show significant differences in ^18^F-FDG uptake (10, 48). Studies comparing FAPI and FDG PET/CT in the same cohort of primary gastric adenocarcinoma patients showed that all primary gastric tumors were FAPI positive (100% detection rate), but that only half of the tumors were FDG positive (50% detection rate) (49). In a study of 38 gastric cancer patients including 31 adenocarcinomas and seven signet-ring cell carcinomas, the sensitivities of ^68^Ga-FAPI-04 PET/CT and ^18^F-FDG PET/CT for the diagnosis of primary gastric cancer were 100% and 82%, respectively. Four cases of adenocarcinoma and three cases of signet-ring cell carcinoma were missed by ^18^F-FDG PET/CT (4). These studies suggested that the FAPI detection rate for primary gastric cancer was better than that of FDG. This may be because of the high intake rate of FDG in the intestinal wall and other abdominal organs (50). Furthermore, the study also found that ^68^Ga-FAPI-04 PET/CT had certain advantages for detecting lymph node metastasis and peritoneal metastasis in gastric cancer (49, 51, 52). Previous studies have shown that ^18^F-FDG PET/CT had a low sensitivity to metastasis in gastrointestinal tumors (53–56). In a subgroup analysis, FAPI outperformed FDG in detection and assessment of the extent of poorly differentiated gastric signet-ring cell carcinoma combined with peritoneal cancer metastasis (57). FDG was particularly poor in this subgroup; in contrast, FAPI was very useful, with higher uptake and minimal or no background activity in diffuse peritoneal metastasis (58). Jiang et al. found that the sensitivities of ^68^Ga-FAPI-04 PET/CT and ^18^F-FDG PET/CT for 10 regional lymph node metastases and distant metastases were 6/10 and 5/10, respectively (4). Guo et al. examined a 63-year-old man, and found enhanced uptake of ^68^Ga-FAPI in the stomach wall and more lesions in the mesentery and omentum than shown on FDG PET/CT. The patient subsequently underwent a histopathological examination to confirm the diagnosis of peritoneal metastasis from gastric adenocarcinoma (59). In a 41-year-old woman recently diagnosed with gastric signet-ring cell carcinoma, ^68^Ga-FAPI PET/CT was performed 2 days after ^18^F-FDG PET/CT. The MIP image of ^68^Ga-FAPI PET/CT showed a significantly higher number of abnormal foci in different parts of the body than that of ^18^F-FDG PET/CT, suggesting gastric signet-ring cell carcinoma had extensive peritoneum, lymph node, and bone metastasis (44). However, some studies have found that inflammation-induced fibrosis may cause false-positive uptake of ^68^Ga-FAPI. In a 78-year-old man who was newly diagnosed with gastric adenocarcinoma, a benign Schmorl node in the inferior endplate of the T5 vertebra showed enhanced uptake of ^68^Ga-FAPI-04, which was not FDG avid. Two months after radical gastrectomy of gastric cancer, a follow-up CT showed that the Schmorl node in the T5 vertebra remained unchanged. This node was suspected to be caused by bone marrow fibrosis and sclerosis after prominent medulla vertebrae (60). In the patients described by Pang et al., false-positive uptake of ^68^Ga-FAPI was observed in those with inflammatory diseases, granulomatous disease, and other diseases where fibrosis was active (16).

**Figure 1:**
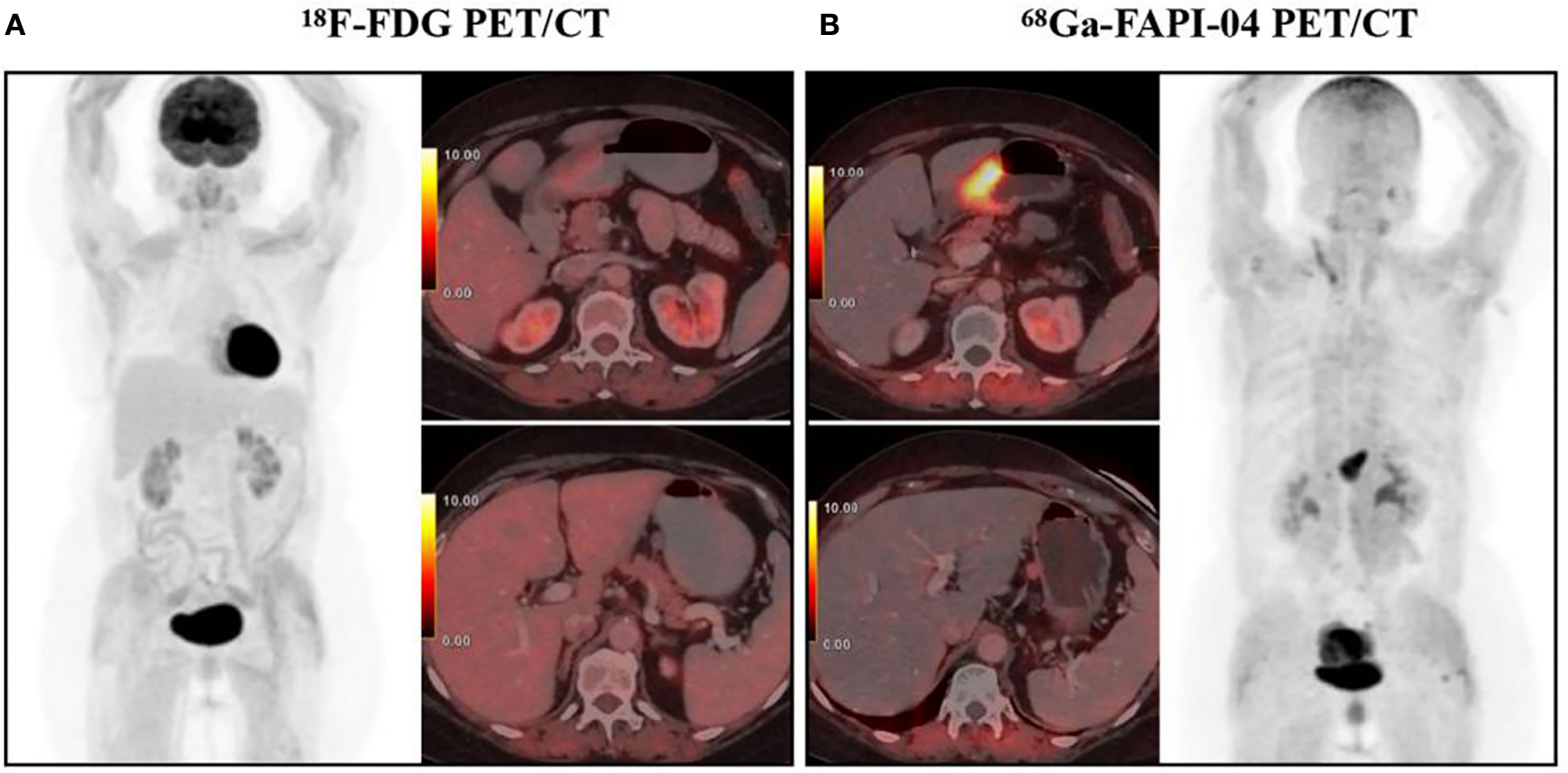
A 65-year-old woman diagnosed with poorly differentiated gastric adenocarcinoma underwent ^18^F-FDG PET/CT **(A)** and ^68^Ga-FAPI-04 PET/CT examination **(B)** respectively, showing a FAPI-positive, FDG-negative primary tumor (SUV_max_ 11.8 and 2.3, respectively) and a perigastric lymph node (SUV_max_ 2.3 and 0.3, respectively). With permission from Kuten et al. (49).

**Figure 2:**
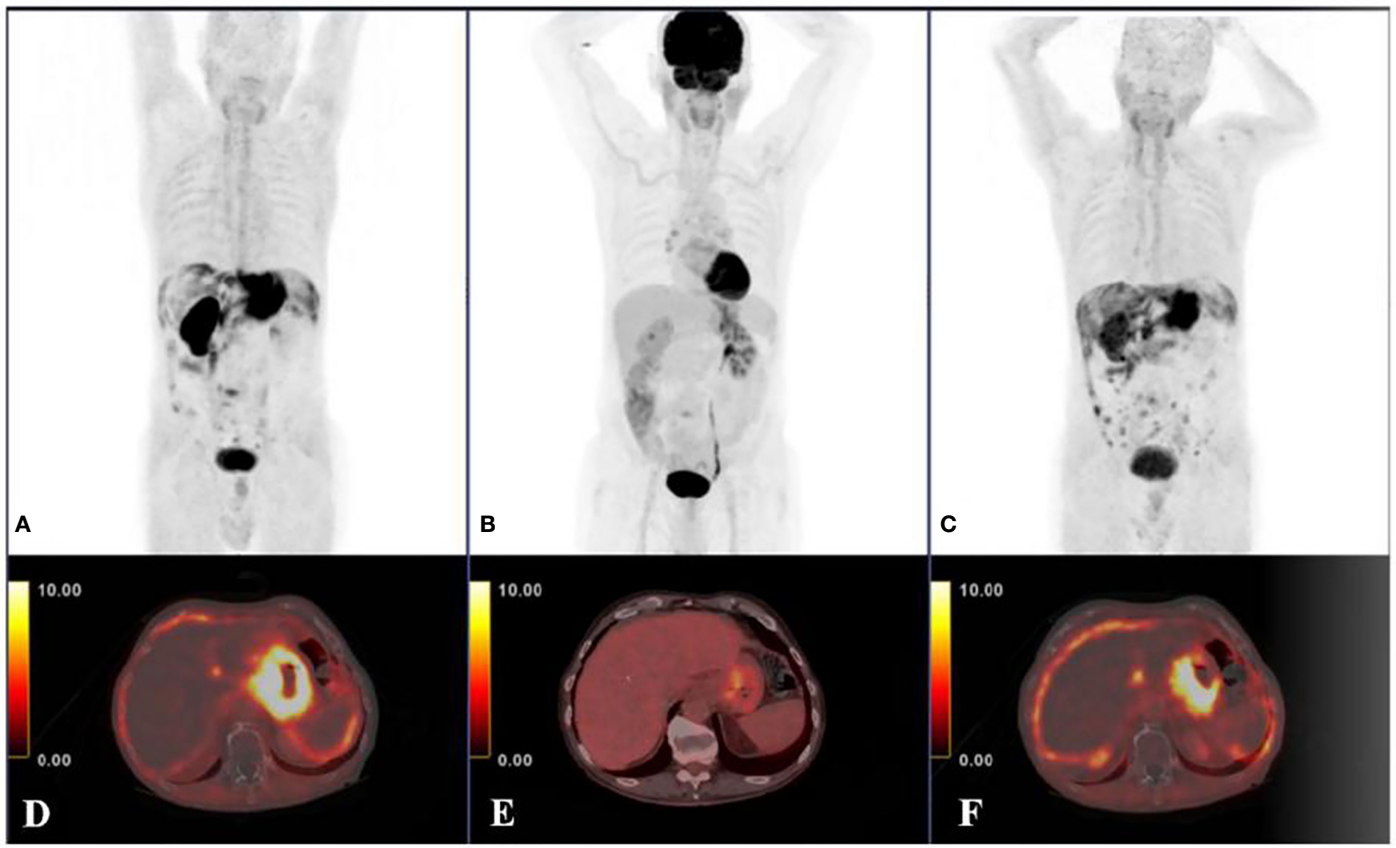
Another 78-year-old male diagnosed with poorly differentiated gastric adenocarcinoma, ^68^Ga-FAPI-04 PET/CT **(A, D)** and ^18^F-FDG PET/CT scans **(B, E)** revealed highly uptake of FAPI in the primary tumor and peritoneal carcinomatosis compared with FDG (primary tumor SUVmax 23 and 6.8; primary tumor tumor-to-background ratio;11.5 and 3.8; peritoneal-carcinomatosis SUVmax 7.5 and 2.3, respectively). The ^68^Ga-FAPI-04 PET/CT examination after 4 months of chemotherapy **(C, F)** showed the disease progression of this patient. The results demonstrated that ^68^Ga-FAPI-04 PET/CT was superior to ^18^F-FDG PET/CT in detecting primary gastric adenocarcinoma and peritoneal carcinomatosis with a gastric cancer origin. With permission from Kuten et al. (49).

These studies indicate that ^68^Ga-FAPI PET/CT has advantages over ^18^F-FDG PET/CT in displaying primary gastric cancer, lymph node metastasis, and peritoneal metastasis of gastric cancer (**Figures 1** and **2**). Largely because of the relatively high physiological background uptake of ^68^Ga-FAPI in the stomach wall and other abdominal organs, it has better tumor-to-background contrast and provides a more detailed profile of the tumor. However, when the tumor invades other tissue, a fibrotic reaction may occur, leading to severe fibrosis and the possible false-positive uptake of ^68^Ga-FAPI. As inflammation may occur concurrently with malignant disease, we should refer to other imaging manifestations and clinical data to avoid misdiagnosis, rather than just using the uptake level of ^68^Ga-FAPI.

Since the studies mentioned here used head-to-head comparison, the imaging protocols used were largely consistent. PET/CT (Biograph mCT, Siemens Healthineers, Germany; Ingenuity TF, Philips Healthcare, USA; uMI510, United Imaging, China, etc.) scanners were used in these studies. The median time between the FDG and the FAPI scans was 1–23 days. The injection dose of each tracer was adjusted by weight (3.7-5 MBq/kg for FDG and 1.8-3 MBq/kg for FAPI-04). Before FDG PET/CT, patients were instructed to fast for at least 4–5h and avoid exercise for 24h. All patients should underwent blood glucose testing to ensure normal blood glucose levels (<150 mg/dl). PET scans were obtained 1h after injection of the radiotracer. Appropriate hydration was ensured before all scanning procedures, and patients were instructed to urinate immediately before imaging acquisition. After low-dose CT scans (tube voltage of 110-120 KeV, current of 80-120 mA, and slice thickness of 3.75 mm), PET scans were collected in 3D mode. Then emission data were corrected, and reconstruction was performed. After correction for the corresponding emission data, the reconstructed images were generated.

#### 2.1.2 Gut

From 1993 to 2013, the incidence of colorectal cancer increased by approximately 2% annually in people younger than 50 years old (6). In 2020, there were over 1.9 million new cases of colorectal cancer (including the anus) and 935 000 deaths, accounting for around one-tenth of cancer cases and deaths. Colorectal cancer ranks third in terms of incidence and second in terms of mortality (1). Because ^18^F-FDG PET/CT has low sensitivity for lymph node staging, it has limited applicability for intestinal tumor staging and surgical planning (61). In a male patient with a biopsy-confirmed peritoneal metastatic adenocarcinoma, ^18^F-FDG PET/CT showed no abnormal abdominal and pelvic activity. On the maximum density projection and axle graph, ^68^Ga-FAPI maximum intensity projection and axial fusion images showed lesions with increased ^68^Ga-FAPI uptake in the peritoneal, mesenteric, omentum, and ileum intestinal wall. ^68^Ga-FAPI PET/CT signs suggested appendix mucinous carcinoma with lymph node metastasis and extensive peritoneal carcinomas (62). Koerber and others performed ^68^Ga-FAPI PET/CT on 22 patients. FAPI tracer had the highest uptake rate in metastatic liver cancer and anal cancer, with maximum specific uptake values (SUV_max_) of 9.1 and 13.9, respectively. In untreated patients, 50% experienced TNM changes. In comparison, 47% of patients developed new findings with metastases (3). In a 23-year-old woman with sigmoid colon signet-ring cell carcinoma, ^68^Ga-FAPI-04 PET/CT showed strong uptake by lesions in the sigmoid colon, retroperitoneal lymph node, left supraclavicular lymph node, and pelvic peritoneum. Compared with ^18^F-FDG, ^68^Ga-FAPI-04 showed clearer and more foci (63). Many studies have confirmed the advantages of ^68^Ga-FAPI for discovering and delineating primary and metastatic cancer foci in colorectal cancer (3, 64, 65). Furthermore, some studies found that ^68^Ga-FAPI had certain advantages for showing inflammation associated with intestinal disease. A 65-year-old woman with rectal cancer showed enhanced ^68^Ga-FAPI uptake in her sacroiliac and costal joints, suggesting that she had sacroiliac arthritis and spondylitis, while ^18^F-FDG PET/CT found no abnormal lesions on the bone (66). MIP images of a 49-year-old male patient showed increased FAPI intake in the middle abdomen, which was followed by a pathological diagnosis of benign enteric inflammatory myofibrolastoma (67). Scholars have also found that FAPI-guided radiation therapy may reduce selective lymph node irradiation, thereby avoiding tumor recurrence and improving overall survival. Preliminary data suggest that as a complementary treatment for conventional radiotherapy, FAPI-guided radiotherapy can significantly improve treatment outcomes (3).

According to previous studies, ^68^Ga-FAPI PET/CT has several advantages over ^18^F-FDG PET/CT in the identification of primary intestinal and metastatic tumors. These are likely due to high levels of fibroblast-activated protein expression in tumors. ^68^Ga-FAPI PET/CT also improves the delineation of target areas in patients receiving radiotherapy. In addition to various malignancies, ^68^Ga-FAPI can be taken up by various non-malignant diseases such as benign enteric inflammatory muscle fibroblastoma and various intestinal inflammations. ^68^Ga-FAPI has been introduced as an imaging radiotracer, which will help to develop new treatment strategies such as FAPI-guided radiotherapy, although more research is needed to confirm the effectiveness of the treatment.

#### 2.1.3 Biliary System

Gallbladder cancer is the most common cancer of the biliary system, ranking among the top six gastrointestinal tumors worldwide (68–71). Gallbladder cancer is not easy to detect in the early stage, and is therefore usually diagnosed in the late stage. It regularly metastasizes and causes biliary obstruction (72). Cholangiocarcinoma is a digestive tumor with a low incidence and poor prognosis (73). It originates from malignant tumors of the bile duct cells arranged on the biliary tree. Anatomically, it can be divided into three subtypes: intrahepatic, perihilar, and distal subtypes. It is a highly malignant tumor with poor prognosis. Approximately 50% of untreated patients die within 3-4 months of developing symptoms (74–76). Very few clinical studies have examined the role of FAPI in the diagnosis of biliary system malignancies. Studies have demonstrated that factors associated with improving the long-term survival of patients with malignancies of the biliary system are the ability to achieve negative surgical margins and the pathological stage (i.e., lymph node metastasis) (77–79). Therefore, it is essential to improve the accuracy of early diagnosis and postoperative review. Kratochwil et al. used ^68^Ga-FAPI PET/CT to evaluate 80 patients with 28 different tumor entities, and found one of the highest SUV values in cholangiocarcinoma (mean SUV_max_ > 12), which showed significantly-high uptake rates and image contrast on FAPI PET/CT (19). Cholangiocarcinoma is a tumor with hyperplastic connective tissue and rich stroma, and the number of CAFs is usually several times that of actual cholangiocarcinoma cells, and thus ^68^Ga-FAPI-04 has high sensitivity for the detection of cholangiocarcinoma (80, 81). Similarly, ^68^Ga-FAPI PET/CT has also been successfully applied to benign lesions of the biliary system. A contrast-enhanced CT scan of a 47-year-old male patient revealed hilar bile duct stenosis with intrahepatic bile duct dilation, and ^18^F-FDG PET/CT showed only mild FDG uptake in the hepatic phylum, whereas ^68^Ga-FAPI PET/CT showed intense radioactivity in the same region of the hepatic portal vein. A final laparotomy resulted in a diagnosis of portal vein biliary lesions caused by cavernous portal degeneration. In this case, the uptake rate of portal cholangiopathy caused by cavernous portal degeneration was higher on ^68^Ga-FAPI PET/CT than on ^18^F-FDG PET/CT (82). A 57-year-old man with a history of colon cancer resection showed enhanced FAPI uptake in the gallbladder wall with a central area of reduced signal on ^68^Ga-FAPI PET/CT. The pathology revealed asymptomatic chronic cholecystitis (83).

### 2.2 Parenchymal Organs

#### 2.2.1 Liver

**Figure 3:**
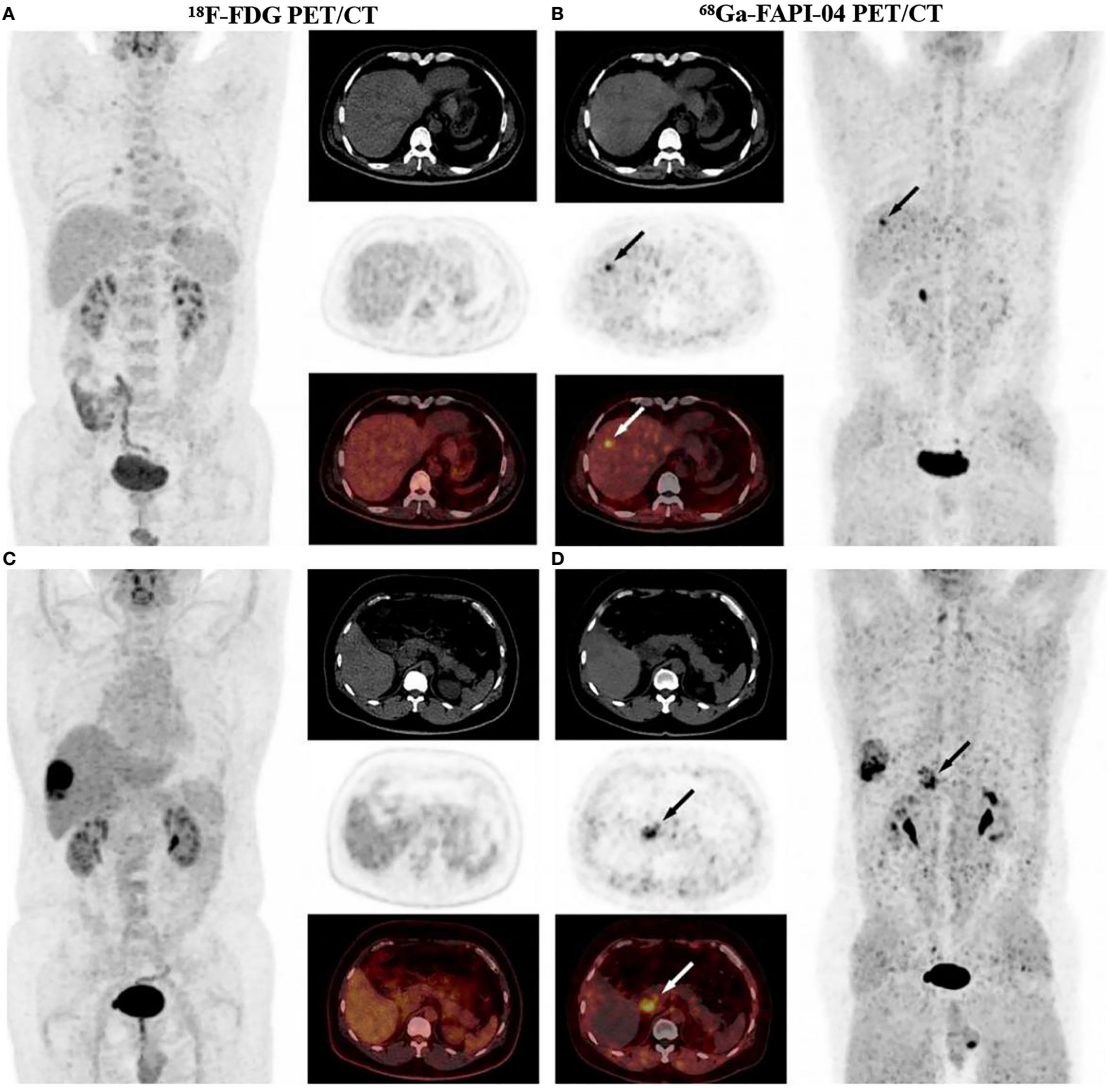
A 53-year-old male diagnosed with moderately-differentiated HCC, ^18^F-FDG PET/CT images did not detect intrahepatic positive findings **(A)**, and ^68^Ga-FAPI-04 PET/CT showed a strongly FAPI positive lesion in the right lobe of the liver **(B)**. In PET/CT images of another 53-year-old male diagnosed I with recurrent HCC and extensive peritoneal diffusion, a small metastatic lesion was not detected in ^18^F-FDG PET/CT **(C)**, which was found with increased uptake in ^68^Ga-FAPI-04 PET/CT **(D)**. Therefore, ^68^Ga-FAPI-04 PET/CT was more sensitive than ^18^F-FDG PET/CT in the detection of HCC and peritoneal metastatic carcinoma. With permission from Wang et al. (45).

In 2020, hepatic cell carcinoma (HCC) was ranked fifth for global male morbidity and second for male mortality. Approximately 906 000 new cases occurred, and the death toll was 830 000 (1). The risk factors for liver cancer include chronic hepatitis B and C, alcohol abuse, metabolic liver disease, and exposure to dietary toxins such as aflatoxin (84, 85). In addition to primary HCC, the incidence of liver metastases is very high. The liver accounts for 75.7% of all synchronous metastatic cancers, and the rate of liver metastasis is higher in men than in women. The prospects for patients with untreated liver metastases are poor with less than 5% survive for five years (2, 86). Early hepatocellular carcinomas are candidates for potential radical treatment, including local ablation, surgical resection, and liver transplantation (87). Therefore, liver cancer monitoring and early detection increase the chance of potential cure (85). Experiments demonstrated that ^68^Ga-FAPI-04 PET/CT has high sensitivity in the detection of primary liver cancer (81, 88–90). Shi et al. detected 28 intrahepatic malignant lesions in 16 patients with suspected liver cancer, with 75% of HCC lesions (n=6) showing significant FAP expression (81). One study has also shown that HCC could be identified from multiple liver tumors using dynamic FAPI PET combined with kinetic modelling, suggesting that dynamic FAPI PET imaging had potential for the accurate non-invasive diagnosis of liver malignancies (91). ^68^Ga-FAPI-04 PET/CT also has advantages in the detection of metastatic liver cancer (**Figure 3**). Sahin et al. set diagnostic criteria for identifying 98 liver metastases in 31 patients with gastrointestinal tumors. Among them, 92 lesions were ^68^Ga-DOTA-FAPI positive, and 65 were ^18^F-FDG positive (92). Another study found that ^68^Ga-FAPI-04 PET/CT showed multiple metastatic liver lesions that were not detected on ^68^Ga-DOTATE PET/CT (93). Another 45-year-old male patient, who had a history of hepatitis A but was cured, underwent a CT scan and found space-occupying lesions in the liver. The patient subsequently underwent ^18^F-FDG PET/CT that showed no abnormalities foundings. The next day, the patient underwent a ^68^Ga-FAPI-04 PET/CT scan, which showed not only strong and high uptake at the tail of the pancreas but also increased uptake in the low background of the liver. Subsequently, postoperative pathology confirmed hepatic metastasis of pancreatic G2 NETs (94). These cases suggest a great potential of ^68^Ga-FAPI-04 in diagnosing liver metastases. However, a 60-year-old female patient with a history of resection of IgG4-related sclerosing cholangitis underwent ^18^F-FDG PET/CT during follow up, and a hypermetabolic lesion with a high-density suture was found on the left edge of the residual liver. Further inspection on ^68^Ga-FAPI PET/CT showed diffuse intense FAPI activity throughout the liver due to histopathological features of “storiform” fibrosis, and ultimately the patient was diagnosed with liver fibrosis on ultrasound transient elastography (95). Kreppel et al. found that tissue remodelling by cirrhosis led to fibroblast activation and FAPI uptake. FAPI PET may be useful for detecting the progression of activity in cirrhosis (96). According to the study, liver metastasis is closely associated with the burden of liver fibrosis, with 80%–90% of liver metastasis occurring in fibrosis or cirrhosis (97). Therefore, the examination results of ^68^Ga-FAPI-04 PET/CT should be comprehensively judged as malignant tumor or fibrosis based on the condition and other auxiliary examinations. Furthermore, FAPI PET/CT may contribute to the planning of radiotherapy for liver tumors because FDG PET has high background signal in the liver, which hinders definition of the target area volume. FAPI PET/CT may also play a role in future checkpoint inhibitor treatment programs for HCC because activated fibroblasts regulate immune cell function in the tumor stroma (89, 96).

#### 2.2.2 Pancreas

The number of deaths from pancreatic cancer (466 000) is almost the same as that of new pancreatic cancer patients (496,000). Pancreatic cancer with a very poor prognosis has been the seventh leading cause of cancer death (1). The risk factors for pancreatic cancer include obesity, diabetes, and excessive alcohol consumption. Because patients rarely show specific symptoms at an early stage, the current early diagnosis of pancreatic cancer depends on imaging (98). PET/CT is more accurate and sensitive for the diagnosis of pancreatic primary and metastatic tumors than contrast-enhanced CT, magnetic resonance imaging (MRI) and MR pancreatic cholangiography (99). The current commonly used clinical examination is ^18^F-FDG PET/CT. However, it has some limitations such as when pancreatic cancer patients are accompanied by hyperglycaemia, in which case ^18^F-FDG can be competitively inhibited resulting in low uptake and the possibility of false negatives. Additionally, ^18^F-FDG PET/CT has low sensitivity and specificity in the diagnosis of pancreatic cancer (100–103), While FAPI PET/CT was found to show high tumor background contrast, and could visualize CAF density and crucial biological information on FAP expression (104). Röhrich et al. performed ^68^Ga-FAPI PET/CT in 19 patients with pancreatic ductal carcinoma, and the ^68^Ga-FAPI PET/CT results altered the TNM stage in 10 out of the 19 patients, resulting in changes in tumor treatment in seven patients (105). Studies were also performed on a 65-year-old male patient with suspected pancreatic cancer using ^18^F-FDG and ^68^Ga-FAPI PET/CT. The uptake intensity of ^68^Ga-FAPI PET/CT was higher than that of ^18^F-FDG in the head of the pancreas and the 10th rib on the right side, and the patient was eventually diagnosed with pancreatic cancer (106). These studies showed that ^68^Ga-FAPI PET/CT was better than ^18^F-FDG PET/CT for diagnosing pancreatic cancer (107). In a male patient considered to have an IgG4-related disease, ^68^Ga-FAPI PET showed inflammation throughout the pancreas and bile duct trees, whereas ^18^F-FDG PET did not. Therefore, ^68^Ga-FAPI is not only a good tumor tracer, but also a good tracer for fibroblast-mediated inflammatory responses (108). However, inflammation-induced non-specific fibrosis may also lead to positive ^68^Ga-FAPI-04 uptake and may thus be mistaken as an indicator of tumor recurrence (109–113). Fortunately, ^68^Ga-FAPI-04 PET/MR has the potential to avoid misdiagnosis of certain pancreatic lesions (114). Fibroblast activator proteins are highly expressed in pancreatic cancer tissues, and most strongly so next to the carcinoma. FAPI has also been taken to establish the radioligand for pancreatic cancer treatment (115, 116). Between June 2020 and March 2021, nine patients with metastatic soft tissue or osteosarcoma (N=6) or pancreatic cancer (N=3) received ^90^Y-FAPI-46 treatment; four of these patients had hepatotoxic side reactions, including the most common one of thrombocytopenia, whereas three patients showed controlled disease with a low incidence of attributable adverse events. This indicated that FAP targeting radiotherapy using ^90^Y-FAPI-46 was well tolerated, and a ^90^Y-FAPI-46 repeat cycle was feasible (117). Studies have also evaluated the biodistribution and theranostic effect of ^225^Ac-and ^64^Cu-labelled FAPI in pancreatic cancer lesions, with ^64^Cu-FAPI-04 dynamic imaging showing rapid renal clearance, slow tumor clearance, and a significant increase in accumulation levels in tumor. ^225^Ac-FAPI-04 showed significant inhibition of tumor growth and no significant effect on body weight. Thus, ^64^Cu-FAPI-04 and ^225^Ac-FAPI-04 can be used as a pair radioligands for theranostic of FAP-expressing pancreatic cancer (118). We also understand that radioligands bearing radionuclides with a short half-life and β^+^ or γ emission could be used for diagnosis, while those with a longer half-life and β^-^ or α decay for therapy (119, 120). However, the clinical data remain sparse, and more extensive trials are needed to determine the efficacy and toxicity of other radionuclides.

#### 2.2.3 Kidney

Renal cell carcinoma (RCC) is the 14th ranked malignant disease worldwide, with a peak incidence at 60-70 years-of-age (121). Other identified risk factors include obesity, smoking, and hypertension (122). Transparent cell carcinoma, papillary carcinoma, and chromophobe renal cell carcinoma (CRCC) are the most common solid RCCs, accounting for 85%-90% of all malignant kidney tumors (123). Because of the low expression of glucose transporter-1 in RCC and the physiological excretion of ^18^F-FDG from the kidney, there may be reduced contrast between kidney disease and normal tissue, which may conceal kidney disease. Therefore, the applications of ^18^F-FDG PET/CT to RCC are limited (9, 13, 14, 124). Pang et al. studied a 71-year-old male patient whose imaging showed multiple ^68^Ga-FAPI active lesions in the cervical and lower thoracic spine, with another high-uptake lesion being observed at the right lower renal pole. Renal biopsy resulted in a diagnosis of CRCC, and ^68^Ga-FAPI PET/CT showed higher activity than ^18^F-FDG in bone metastases (125). In addition to kidney cancer, chronic kidney disease is clinically prevalent. Risk factors for chronic kidney disease include diabetes, hypertension, obesity, and old age. Current clinical treatments can only delay the progression of disease, and may ultimately lead to irreversible nephron loss and end-stage renal disease (ESRD) (126). Kidney fibrosis is a pathological state occurring during the progression of chronic kidney disease, and a strategy targeting activated myofibroblasts and collagen-degrading enzymes may decrease or even reverse kidney fibrosis (127). Zhou et al. performed ^68^Ga-FAPI-04 PET/CT imaging on 13 patients with renal fibrosis, and the examinations indicated that almost all patients (12/13) showed increased radiotracer uptake. The SUV_max_ of the patients with mild, moderate, and severe fibrosis were 3.92 ± 1.50, 5.98 ± 1.6 and 7.67 ± 2.23, respectively. Thus, in comparison with renopuncture examinations, ^68^Ga-FAPI-04 PET/CT showed bilateral kidneys more quickly and sensitively, which can, in turn, help assess disease progression, diagnosis, and treatment planning (67).

## 3 Pelvic Organs

### 3.1 Uterus

The most common malignant tumors in the uterus are those from cervical cancer and uterine body cancer. Cervical cancer is the fourth most commonly diagnosed cancer and the fourth leading cause of cancer death in women, with an estimated 604 000 new cases globally and 342 000 deaths in 2020 (1). Human papillomavirus (HPV) is the primary cause of cervical cancer. Cervical cancer is a preventable disease for which screening tests can be used to detect precancerous lesions (128, 129). Body cancer (mainly endometrial cancer) ranks sixth most common among women worldwide. There were 417 000 new cases in 2020 and 97 000 deaths (1). The risk factors for endometrial cancer include early menarche, late menopause, infertility, and obesity (130). Studies have found that the endometrium has a physiological uptake of FDG, which may lead to a false-positive or false-negative diagnosis. A 44-year-old woman with newly diagnosed gastric adenocarcinoma by gastroscopy underwent ^18^F-FDG PET/CT, which showed strong uptake in the uterine area. Physiologic uptake of endometrium was initially suspected but eventually proved to be mucinous adenocarcinoma originating from the stomach (131). In addition, there was also a physiological uptake of FAPI in the endometrium. Dendl et al. assessed the biodistribution and tumor uptake of multiple gynaecological tumors. In eight primary tumors, the uptake rate of all primary lesions was highest in endometrial cancer (mean SUV_max_ = 18.4), followed by cervical cancer (mean SUV_max_ = 15.22). However, in studies of normal hormone-responsive organs, there was a significant difference in the uptake of the endometrium (11.7 vs 3.9; p<0.0001) and breast (1.8 vs 1.0; p=0.004) between premenopausal and postmenopausal patients; therefore, increased endometrial FAPI uptake does not necessarily imply tumor infiltration into the uterus. FAPI accumulates in the tissue during remodelling, suggesting that the postmenopausal endometrium remains active and in a quiescent state, rather than truly atrophic (132). This was further evidenced by Dendl et al. in another study on a 29-year-old female patient with adenoid cystic carcinoma. In addition to the high uptake of ^68^Ga-FAPI-46 by the adenoid cystic carcinoma, the parenchyma of both breasts was well depicted, with SUV_max_ of 4.1 on the right and 3.5 on the left. The SUV_max_ of the endometrium was 25.7. This case suggests that the expression of FAP in hormone-sensitive organs such as the breast and uterus may undergo a physiological increase after pregnancy and during lactation (133). The latest study by Christine E. Mona et al. showed that the highest ^68^Ga-FAPI-46 PET signal in the normal organ was the bladder due to the urinary bladder due to urinary excretion and the uterus due to normal myometrial FAP expression (134).

Experimental data show that FAPI can increase physiologically in the uterus during normal pregnancy and lactation, and that this can affect the clinical discrimination of uterine malignancies. However, there is currently little clinical research on the application of FAPI in malignant uterine tumors. The question of how to overcome the impact of false-positive tumor uptake still requires clinical trials and innovative ideas.

### 3.2 Ovary

**Figure 4:**
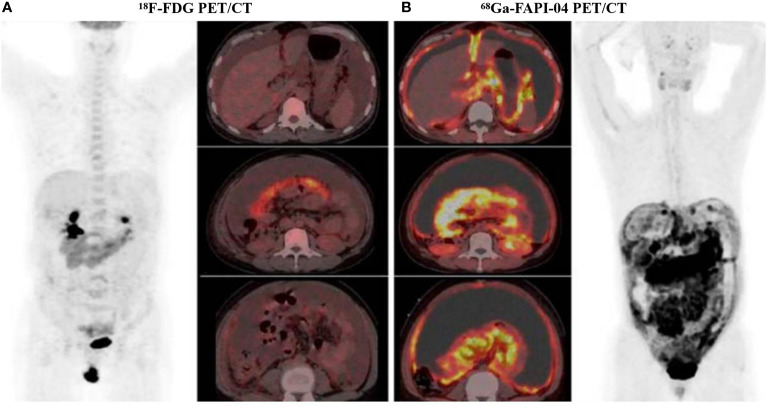
A 47-year-old woman diagnosed with ovarian cancer, ^18^F-FDG PET/CT showed the omental-cake-pattern of peritoneal carcinomatosis with low-to-moderate activity throughout the entire abdomen and pelvis **(A)**. However, there were more details in ^68^Ga-FAPI-04 PET/CT images, we could find that higher uptake was observed in the omental and peritoneal surfaces, especially around the liver and small bowel mesenterium **(B)**. Subsequent pathological findings were consistent with ^68^Ga-FAPI-04 PET/CT findings. ^68^Ga-FAPI-04 PET/CT showed superiority over ^18^F-FDG PET/CT in the display of peritoneal metastatic carcinoma. With permission from Chen et al. (88).

Ovarian cancer is a significant cause of cancer morbidity and mortality in women, and it is an aggressive epithelial tumor (135). Integrated treatment by gynaecologists, medical oncologists, pathologists, and radiologists is essential to improve the prognosis of ovarian cancer (136). One study found that CAF was associated with the stage of epithelial ovarian cancer, lymph node metastasis, and omental metastasis (137). ^18^F-FDG PET/CT can be used to detect the early recurrence of ovarian cancer before lesions are observable on CT or the onset of clinical symptoms, especially in patients with elevated CA-125 levels. However, ^18^F-FDG accumulated in the gut and was then excreted through the urinary tract. It may therefore interfere with the optimal assessment of primary pelvic tumors (11). ^18^F-FDG PET/CT had some limitations when used for the detection of primary ovarian cancer tumors and delineating masses for pre-treatment plans, which may have implications for treatment approaches (138). FAP is overexpressed on the CAFs of ovarian cancer (32, 139), and a study showed that the SUV_max_ of ^68^Ga-FAPI-04 for ovarian cancer was moderate (6–12), while FAPI showed low non-specific intestinal/peritoneal uptake rates. This may mean a better diagnosis of peritoneal cancer because peritoneal cancer is the primary clinical challenge of advanced ovarian cancer (**Figure 4**) (19). At the time of the literature study for this review, no other teams had applied FAPI in ovarian cancer. More clinical studies are needed to confirm whether FAPI has advantages in the diagnosis and treatment of ovarian cancer.

### 3.3 Prostate

Prostate cancer is the second most common cancer in men and is the fifth most common cause of death from cancer. In 2020, there were nearly 1.4 million new cases of prostate cancer worldwide and 375 000 deaths (1). A total of 93% of prostate cancers are adenoid adenocarcinomas, while the remaining 7% are variants of ductal adenocarcinoma, basal cell carcinoma, and neuroendocrine tumors (140). Recent findings indicate that ^177^Lu-PSMA (prostate specific membrane antigen) may provide an effective treatment option for patients with high expression of PSMA (141). Although PSMA-targeted imaging probes have been used for prostate cancer diagnosis, variable expression in metastases and difficulties in detecting visceral lesions have limited their application (142). Three male patients with advanced castration-resistant prostate cancer or neuroendocrine prostate cancer underwent ^68^Ga-FAPI-04 PET/CT examinations. The results revealed that FAP expression increased significantly with disease progression. In patients with advanced castration-resistant prostate cancer, ^68^Ga-FAPI-04 PET/CT was highly positive (143). Other researchers found that FAP expression is significantly increased in metastatic disease compared with primary prostate cancer (144). However, in a study of a 76-year-old man with a history of chronic prostatitis and left shoulder osteoarthritis, ^18^F-FDG and ^68^Ga-DOTA-FAPI-04 PET/CT examinations were similar, indicating that for prostate cancer, ^68^Ga-DOTA-FAPI-04 imaging may not be more tumor-specific than ^18^F-FDG imaging. The study also found that the shoulder arthritis site showed higher tracer uptake than the tumor site. This revealed that ^68^Ga-DOTA-FAPI-04 imaging was beneficial for diagnosing inflammation, but that inflammation can cause substantial interference in the diagnosis of tumors.

Combining the information from the above studies, we found that the application value of ^68^Ga-FAPI-04 imaging in prostate cancer is uncertain. The potential value of ^68^Ga-FAPI-04 imaging in inflammation may reduce its diagnostic specificity in tumors. Is ^68^Ga-FAPI-04 PET/CT more tumor-specific than ^18^F-FDG imaging in prostate cancer, or does it have more diagnostic and therapeutic effects? Does FAPI-targeted radionuclide therapy have potential? These questions require further clinical studies and more extensive clinical data.

## 4 Conclusion

In this review, we describe the current status of FAPI PET/CT in abdominal and pelvic organ tumors, and list many corresponding false-positive cases. Current clinical studies demonstrate that molecular imaging with FAPI PET/CT has good prospects for applications in abdominal and pelvic tumors. It has advantages over ^18^F-FDG PET/CT in the diagnosis of primary tumors and various metastases. Currently, FAPI-02 and FAPI-04 were the commonly used ligands to synthesize imaging agent. Previous studies have shown that FAPI-04 had a longer half-life than FAPI-02, and had excellent stability in human serum. In the lesions, FAPI-04 exhibited longer radiotracer residence. Compared to the effective tumor uptake of FAPI‐02, the effective tumor uptake of FAPI‐04 after 24 hours increased by a hundred percent, which had significant benefits for therapeutic application of the tracer. Therefore, FAPI-04 is more advantageous. However, there are currently little clinical data on the application of FAPI in malignant tumors of pelvic organs such as the uterus, ovary, and prostate, and false-positive results induced by inflammation may limit the application of FAPI PET/CT. These problems also require systematic clinical studies with large patient populations.

Tumor therapeutic strategies using FAPI are also worth exploring. The low expression of FAP in normal tissues is a significant advantage of targeted therapy with FAPI. For future studies, radionuclides with suitable half-life and β- or α decay may be the best option for radiotherapy. Combined with chemotherapy and immunotherapy, it may produce synergistic results. Moreover, radiotherapy causes biological and molecular changes in the tumor microenvironment, which can be observed by PET tracers. The plan can be adjusted during treatment, and the subsequent treatment plan can be personalized with this information. The application of FAPI PET/CT to tumor evaluation and restaging has been shown to be up-and-coming, but the research is still in its infancy. With the deepening of research and the constant maturity of technology, we believe that FAPI PET/CT will be more widely used in clinical practice.

## Author Contributions

TY collected the literature and wrote the manuscript. LM and HH offered methodology for the review. WT and FG organized thoughts and revised the review. All authors contributed to the article and approved the submitted version.

## Funding

This study was funded by Huai’an Science and Technology Project (grant no. HAB202017 to WT), High-Level Talent Funding Project of the College (grant no. YGRX201906 to WT).

## Conflict of Interest

The authors declare that the research was conducted in the absence of any commercial or financial relationships that could be construed as a potential conflict of interest.

## Publisher’s Note

All claims expressed in this article are solely those of the authors and do not necessarily represent those of their affiliated organizations, or those of the publisher, the editors and the reviewers. Any product that may be evaluated in this article, or claim that may be made by its manufacturer, is not guaranteed or endorsed by the publisher.
